# Neurohemodynamic correlates of ‘OM’ chanting: A pilot functional magnetic resonance imaging study

**DOI:** 10.4103/0973-6131.78171

**Published:** 2011

**Authors:** Bangalore G Kalyani, Ganesan Venkatasubramanian, Rashmi Arasappa, Naren P Rao, Sunil V Kalmady, Rishikesh V Behere, Hariprasad Rao, Mandapati K Vasudev, Bangalore N Gangadhar

**Affiliations:** Department of Psychiatry, Advanced Center for Yoga, National Institute of Mental Health and Neurosciences, Bangalore – 560 029, India

**Keywords:** Meditation, fMRI, ‘OM’ chanting, vagus nerve stimulation

## Abstract

**Background:**

A sensation of vibration is experienced during audible ‘OM’ chanting. This has the potential for vagus nerve stimulation through its auricular branches and the effects on the brain thereof. The neurohemodynamic correlates of ‘OM’ chanting are yet to be explored.

**Materials and Methods:**

Using functional Magnetic Resonance Imaging (fMRI), the neurohemodynamic correlates of audible ‘OM’ chanting were examined in right-handed healthy volunteers (*n*=12; nine men). The ‘OM’ chanting condition was compared with pronunciation of “ssss” as well as a rest state. fMRI analysis was done using Statistical Parametric Mapping 5 (SPM5).

**Results:**

In this study, significant deactivation was observed bilaterally during ‘OM’ chanting in comparison to the resting brain state in bilateral orbitofrontal, anterior cingulate, parahippocampal gyri, thalami and hippocampi. The right amygdala too demonstrated significant deactivation. No significant activation was observed during ‘OM’ chanting. In contrast, neither activation nor deactivation occurred in these brain regions during the comparative task – namely the ‘ssss’ pronunciation condition.

**Conclusion:**

The neurohemodynamic correlates of ‘OM’ chanting indicate limbic deactivation. As similar observations have been recorded with vagus nerve stimulation treatment used in depression and epilepsy, the study findings argue for a potential role of this ‘OM’ chanting in clinical practice.

## INTRODUCTION

Vagal nerve stimulation (VNS) is used as treatment in depression and epilepsy.[1,2] A positron emission tomography (PET) study[3] has shown decreased blood flow to limbic brain regions during direct (cervical) VNS. Another functional magnetic resonance imaging (fMRI) study[4] has shown significant deactivation of limbic brain regions during transcutaneous VNS. In this procedure electrical stimulus is applied over the inner part of the left tragus and hence the auricular branch of the vagus.

The use of ‘OM’ chanting for meditation is well known.[5] Effective ‘OM’ chanting is associated with the experience of vibration sensation around the ears. It is expected that such a sensation is also transmitted through the auricular branch of the vagus nerve. We therefore hypothesized that like transcutaneous VNS, ‘OM’ chanting too produces limbic deactivation. Specifically, we predicted that ‘OM’ chanting would evoke similar neurohemodynamic correlates, deactivation of the limbic brain regions, amygdala, hippocampus, parahippocampal gyrus, insula, orbitofrontal and anterior cingulate cortices and thalamus) as were found in the previous study.[4]

## MATERIALS AND METHODS

Healthy volunteers (*n*=12; nine men) who were right-handed and were consenting to participate as controls in an ongoing MRI research were approached. Two qualified psychiatrists independently assessed these volunteers to exclude: 1) Psychiatric diagnosis, 2) family history of major psychiatric disorder in first-degree relative, 3) pregnancy or post-partum, 4) co-morbid substance abuse or dependence, 5) significant neurologic disorder, 6) any contraindication for MRI and, 7) left/mixed handedness. The absence of psychiatric diagnosis was established using Mini International Neuropsychiatric Interview Plus.[6] The age range of the subjects was 22-39 years (mean±SD=28±6 years). All were literate. Four of these had formal training in yoga including meditation and the rest were naïve to this technique. The NIMHANS ethics committee had cleared the experimental protocol. In addition to the consent that they had already given for the ongoing imaging study they were provided with additional information about the present research (fMRI) and the need to be trained to chant ‘OM’ prior to the fMRI test. Written consent was obtained from all subjects for this study.

### fMRI task

All the subjects were trained in ‘OM’ chanting by an experienced yoga teacher. The subjects were trained to chant ‘OM’ without distress and interruption – the vowel (O) part of the ‘OM’ for 5 sec continuing into the consonant (M) part of the ‘OM’ for the next 10 sec. While earlier electrophysiological studies used mental ‘OM’ chanting, loud chanting of ‘OM’ was chosen in this study. This helped to objectively confirm the task performance during fMRI as well as to provide the vibration sensation and stimulate vagus nerves via the auricular branches thereof. The control condition was continuous production of ‘sssss….’ syllable for the same duration (15 sec). This was chosen to control for the expiratory act of chanting ‘OM’ but without the vibratory sensation around the ears. These practices were achieved in a supine posture. They were familiarized with the same procedure while lying in the MRI console. Once they were comfortable, the fMRI procedure was conducted. At the end of the task, one of the investigators ascertained if the subjects experienced a vibration sensation while chanting ‘OM’ but not “ssss”.

**Figure 1:**
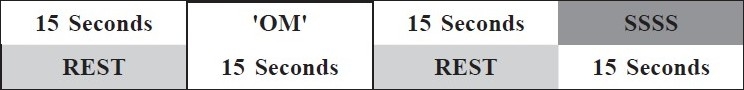
Shows one cycle of REST-‘OM’-REST-ssss; 10 such cycles were performed by each subject during the fMRI scan

The fMRI procedure had a block design. The fMRI experiment consisted of the following phases: 1) a high-resolution structural brain scan was first performed; 2) this was followed by echoplanar imaging (EPI) sequence in which blood oxygen level-dependent (BOLD) scans were performed. The EPI scans had a repetition time (TR) of 3 sec. Two hundred EPI scans were performed over 10 min. These 10 min consisted of 15-sec blocks of ‘OM’ and “ssss”. These blocks were interspersed with 15 sec of rest period. Altogether there were 10 blocks of ‘OM’, 10 blocks of “ssss” and 20 blocks of rest Figure 1.

### Image sequences

Imaging was done using 3 Tesla MRI scanner at NIMHANS. After the initial localization sequences, high-resolution T_1_-weighted, structural MR images of 1-mm slice thickness with no inter-slice gap were obtained (TR=8.1 msec; TE=3.7 msec; matrix=256×256). This high-resolution structural image was utilized for the purpose of localization of brain activation and also to rule out any gross brain abnormality in the study subjects. This was followed by a BOLD sensitive EPI sequence (TR=3000 msec; TE=35 msec; slice thickness=8 mm; number of slices=16; matrix=128×128). The total duration of EPI scans was 10 min. During the EPI scans, the subjects were cued to alternate among various states (i.e., ‘OM’, “ssss” and “REST”) every 15 sec (as described above) through a MRI-compatible monitor display which was synchronized with the image acquisition by e-prime software incorporated in eloquence fMRI hardware setup.

### Image analysis

fMRI analyses were carried out for all patients using Statistical Parametric Mapping 5 (SPM5) (http://www.fil.ion.ucl.ac.uk/spm). Images were realigned, corrected for slice timing variations, spatially normalized[7–9] and smoothened with a Gaussian kernel of 8-mm full-width-at-half-maximum. The blocks were modeled by a canonical hemodynamic response function. SPM5 combines the General Linear Model and Gaussian random field theory to draw statistical inferences from BOLD response data regarding deviations from the null hypothesis in three-dimensional brain space. The voxel-wise fixed effects analysis produced a statistical parametric map in the stereotactic space of the Montreal Neurological Institute[10]. ‘OM’ as well as “ssss”-related BOLD-activation and de-activation, were assessed using a subtraction paradigm by respectively contrasting with the “REST” condition. The BOLD changes were examined specifically in the *à priori* regions-of-interest, namely the limbic brain regions [amygdala, hippocampus, parahippocampal gyrus, insula, orbitofrontal and anterior cingulate cortices and thalamus – the last three brain regions were examined because of their intricate connections with the limbic brain]. For these à priori regions-of-interest masks were created using the WFU Pickatlas for SPM analyses.[11] Significance corrections for multiple comparisons for the individual region-of-interest were performed using a Family-wise Error Correction (FWE) [*P*<0.001].

## RESULTS

**Figure 2:**
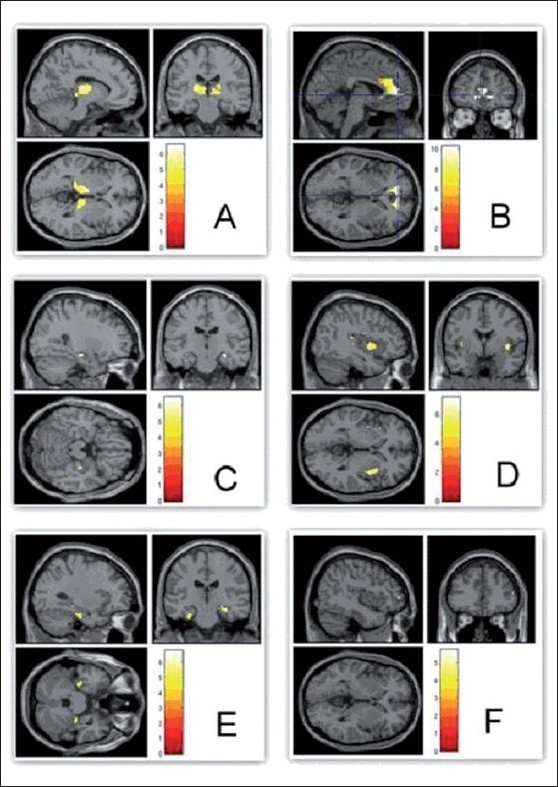
Compared to REST, ‘OM’ chanting produced deactivation of thalami (A) and limbic structures - anterior cingulum (B), hippocampi (C), insula (D) and parahippocampi (E); Whereas control condition ‘ssss’ produced no deactivation in any of these regions (F). The color bar represents the T scores given in the table

Compared to rest condition the BOLD fMRI signals did not detect any significant brain activation during ‘OM’ chanting. However, significant deactivation was seen in the amygdala, anterior cingulate gyrus, hippocampus, insula, orbitofrontal cortex, parahippocampal gyrus and thalamus during ‘OM’ chanting [Table 1 and Figure 2]. The “ssss” task did not produce any significant activation/deactivation in any of these brain regions. The coordinates of significant areas of deactivation were transformed from MNI space[10] into the stereotactic space of Talairach and Tournoux.[12]

**Table 1:** Brain regions with significant deactivation during ‘OM’ condition in comparison with “REST” condition

Brain region**	X	Y	Z	T	FWE-p*
Right amygdala	24	-10	-08	5.2	<0.001
Left anterior cingulate gyrus	-02	45	-02	10.2	<0.001
Right anterior cingulate gyrus	12	49	-01	9.8	<0.001
Left hippocampus	-32	-18	-11	6.5	<0.001
Right hippocampus	30	-31	-05	4.6	<0.001
Left insula	-28	19	-06	6.5	<0.001
Right insula	38	15	-06	4.9	<0.001
Left orbitofrontal cortex	-28	29	-08	6.6	<0.001
Right orbitofrontal cortex	30	29	-08	7.3	<0.001
Left parahippocampal gyrus	-30	-20	-21	5.1	<0.001
Right parahippocampal gyrus	32	-28	-22	5.0	<0.001
Left thalamus	-14	-05	13	6.6	<0.001
Right thalamus	16	-07	11	6.2	<0.001

** X, Y, Z Talairach coordinates of peak activation

* Family-wise error corrected *‘P’* value

## DISCUSSION

In this study, significant deactivation was observed bilaterally during ‘OM’ chanting in comparison to the resting brain state in orbito-frontal, anterior cingulate, parahippocampal gyri thalami and hippocampi. In addition the right amygdala demonstrated significant deactivation. No significant activation was observed during ‘OM’ chanting. In contrast, neither activation nor deactivation occurred in these brain regions during the comparative task – namely the ‘ssss’ condition.

Though there is no previous report on the effect of ‘OM’ chanting on brain hemodynamic responses, an earlier study by Kraus *et al*.,[4] had examined the impact of transcutaneous VNS on BOLD changes using fMRI. Because of the commonality of the vagus involvement (as hypothesized in the current study), we compared our study observations with this earlier study.[4] Interestingly, our study findings are in tune with this previous study; significant deactivation was observed in the amygdala, parahippocampal, hippocampal brain regions. This suggests that neurophysiological effects of ‘OM’ chanting may be mediated through the auricular branches of the vagal nerves. Using a different methodology (positron emission tomography), other researchers[3] demonstrated reduced blood flow bilaterally in the hippocampus, amygdala, and cingulate gyri following left cervical VNS in epilepsy patients. Similarly, VNS treatment in depressed patients reduced regional cerebral blood flow in the amygdala, left hippocampus, left subgenual cingulate cortex, left and right ventral anterior cingulum, right thalamus and brainstem as measured by single photon emission computed tomography.[13] Interestingly, these regions become hyperactivated in patients with depressive disorder[14] for which VNS is used as therapy. However, our observations to support VNS as the mechanism of ‘OM’ chanting are preliminary and further studies are required to support our hypothesis.

Alternatively, ‘OM’ chanting may have been a cue to relaxation. As meditation is shown to activate structures involved in relaxation response, namely cingulate cortex, dorsolateral, prefrontal and parietal cortices, hippocampus and temporal lobes,[15] the confounding effect of relaxation could not be ruled out.

In summary, the hemodynamic correlates of ‘OM’ chanting indicate limbic deactivation. Since similar observations have been recorded with VNS treatment used in depression and epilepsy, the clinical significance of ‘OM’ chanting merits further research.
